# The impact of lipid metabolism on breast cancer: a review about its role in tumorigenesis and immune escape

**DOI:** 10.1186/s12964-023-01178-1

**Published:** 2023-06-27

**Authors:** Diandra Zipinotti dos Santos, Josiany Carlos de Souza, Tatiana Massariol Pimenta, Bárbara da Silva Martins, Roberto Silva Ribeiro Junior, Solenny Maria Silva Butzene, Nayara Gusmão Tessarolo, Paulo Morais Lyra Cilas, Ian Victor Silva, Leticia B. A. Rangel

**Affiliations:** 1 Biotechnology Program/RENORBIO, Health Sciences Center, Federal University of Espírito Santo, Vitoria (Espírito Santo), Brazil; 2 Department of Pharmaceutical Sciences, Federal University of Espirito Santo, Marechal Campos Avenue, MaruípeEspírito Santo, Vitória 1468 Brazil; 3 Viral Vector Laboratory, Center for Translational Investigation in Oncology, Cancer Institute of São Paulo/LIM24, University of São Paulo School of Medicine, São Paulo, (São Paulo), Brazil; 4 Moffitt Cancer Center, Tampa, US; 5 Department of Morphology, Health Sciences Center, Federal University of Espirito Santo, Vitoria, Espirito Santo Brazil; 6 Biochemistry Program, Health Sciences Center, Federal University of Espirito Santo, Vitoria, Brazil

**Keywords:** Lipid metabolism, Immune escape, Tumorigenesis, Breast cancer

## Abstract

**Background:**

Breast cancer (BC) is the second most frequent type of cancer in the world and most common among women, configuring a major challenge to global health. BC is a complex and heterogeneous disease that can be subdivided into distinct tumor types based on the expression of molecular markers predicting patient outcomes and response to therapy. A growing number of studies have tried to expand the known markers by investigating the association of altered lipid metabolism with BC immune escape, progression, and metastasis. In this review, we describe the metabolic peculiarities of each BC subtype, understanding how this influences its aggressiveness and identifying whether these intrinsic vulnerabilities of each subtype can play a role in therapeutic management and may affect immune system cells in the tumor microenvironment.

**Conclusion:**

The evidence suggests so far that when changes occur in lipid pathways, it can affect the availability of structural lipids for membrane synthesis, lipid synthesis, and degradation that contribute to energy homeostasis and cell signaling functions. These findings will guide the next steps on the path to understanding the mechanisms underlying how lipids alterations are related to disparities in chemotherapeutic response and immune escape in BC.

Video Abstract

**Supplementary Information:**

The online version contains supplementary material available at 10.1186/s12964-023-01178-1.

## Background

Breast cancer (BC) is the second most common cancer worldwide and the fifth leading cause of cancer-related death in 2020 among women [1]. Despite advances in diagnosis and treatment, a significant number of patients experience relapse after initial treatment and develop chemoresistance, leading to poor prognosis, lower overall survival rates, and decreased quality of life [2, 3]. This poses a significant public health concern, highlighting the need for effective therapies that prevent progression or treat BC. Perhaps this is the time to broaden the investigation for non-canonical strategies, as well as novel clinical approaches to fight the disease.

The devastating epidemiological profile of BC, in general, reflects, at least partially, the limited knowledge regarding the molecular signaling pathways of tumorigenesis and the function of numerous genes in cancer [4, 5]. In this context, it is crucial to continuously advance our understanding and application of new treatment protocols for the benefit of cancer patients and society. Therefore, comprehending the signaling pathways involved in the development, initiation and immune escape of BC may unveil potential therapeutic strategies and new targets for the disease.

Several risk factors have been associated with BC, including genetic predisposition, lifestyle choices, and environmental factors. These factors include advanced age for first pregnancy, alcohol consumption, physical inactivity, and obesity. Obesity has become a prevalent ailment in contemporary life, characterized by an increase in fat mass due to an energy imbalance. Substantial evidence has implicated obesity as a risk factor for various cancers, including BC [6, 7]. Numerous studies in both animals and humans have demonstrated the impact of obesity on BC phenotype and the pathological role of lipids in the disease. The association between being overweight and BC was initially reported in 1976, where obese BC patients exhibited significantly larger and more invasive tumors, along with a 25.3% lower survival rate compared to the control group [8]. Since then, an extensive investigation has been underway to gain a deeper understanding of the correlation between the lipid profile of patients and BC.

A retrospective study involving 5,683,000 patients in clinical trials with BC undergoing systemic adjuvant chemotherapy based on anthracyclines and taxanes, clearly demonstrated the correlation between obesity and worse clinical outcomes in obese patients compared to non-obese ones [9]. Patients with obesity exhibited higher rates of recurrence (hazard ratio = 1.26, *p* = 0.048) and mortality (hazard ratio = 1.35, *p* = 0.016) compared to non-obese patients. Another retrospective study involving 4,077 women with estrogen receptor (ER) positive BC undergoing various chemotherapy treatments further supported this association. The overall mortality rate was higher among obese patients compared to non-obese patients (hazard ratio = 1.31, 95% confidence interval = 1.12 to 1.54) [10]. Moreover, numerous studies have investigated the relationship between obesity and metabolic alterations with different BC subtypes. Agresti and colleagues (2016) found that a large waist circumference (≥ 80 cm) was associated with an increased risk of developing the luminal B BC subtype. Additionally, women with a body mass index (BMI) higher than 25 kg/m2 had an elevated risk of developing the basal-type triple-negative breast cancer (TNBC) compared to those with a lower BMI [11].

While much attention has been given to obesity, studies are also exploring changes in lipid metabolism within tumor cells to understand their association with prognosis and treatment efficacy. Maiti et al. [12] conducted a study revealing that dyslipidemia, strongly linked to obesity, may also contribute to poor outcomes in TNBC patients. Triglycerides serve as an independent source for fatty acids (FAs) oxidation, which plays a critical role in promoting cell proliferation and tumor growth [13], suggesting the potential carcinogenicity of imbalanced lipid levels.

Furthermore, lipid imbalance can lead to various changes such as tumor-associated macrophages (TAMs) activation in the tumor microenvironment (TME) that further promote tumor progression [14]. Tumor-infiltrating dendritic cells (TIDCs) play a crucial role in stimulating antitumor T cells. In an animal model**,** female C57BL/6 mice and athymic C57 nude mice, Jiang et al. [15] observed a relationship between increased lipid levels in tumor-infiltrated dendritic cells (TIDCs) and the immunosuppression of antitumor T cells. These findings indicate a connection between lipid metabolism, the tumor microenvironment, and the aggressiveness of BC, highlighting their relevance for further research. Recently, several studies have reported a correlation between dyslipidemia and an increased risk of TNBC progressing to a worse condition. However, no such correlation seems to exist for patients with estrogen receptor (ER)-negative and progesterone receptor (PR)-positive BC [11, 16–23] Based on the analysis of the current available data, there is evidence supporting the hypothesis that gaining a better understanding of the role of lipids in the development of BC can lead to the implementation of supportive strategies in the clinical management of patients with this disease.

### The essence of lipids

Lipids play a crucial role as essential components of the body's physiological system. According to the "Comprehensive Classification System for Lipids" published in 2005, lipids are hydrophobic or amphipathic small molecules formed through carbanion-based condensations of thioesters and/or carbocation-based condensations of isoprene units [24]. They can be categorized into eight groups: FAs, glycerolipids, glycerophospholipids, sphingolipids, saccharolipids, polyketides, sterol lipids, and prenol lipids. The functionalities of individual lipids are inherently interconnected with the synergistic behavior of lipid assemblies. The effectiveness of these assemblies greatly relies on their unique lipid composition and the dynamic modifications it undergoes within specific subcellular sites. FAs and cholesterol are emerging as novel regulators of many of these processes.

The body obtains FAs through endogenous synthesis, primarily occurring in the liver, adipocytes, and lactating breast tissues [25]. Additionally, exogenous sources provide FAs through dietary intake as free FAs or as complexed proteins such as low-density lipoproteins [26]. Generally, normal cells rely more on exogenous sources, with limited endogenous FAs synthesis. Unused neutral lipids can be stored in intracellular structures known as lipid droplets. When in action, lipid molecules serve various biological functions within cells. For example, triacylglycerides are used for energy storage, while phosphoglycerides, sterols, and sphingolipids contribute to the structural components of cellular membranes. Lipids also act as important metabolic signaling messengers and hormones [27] such as estrogen, progesterone [28, 29] and testosterone [30]. The role as second messenger can be performed by diacylglycerol [31], phosphatidic acid [32], and sphingolipids including ceramide-1-phosphate, sphingosine (Sph), Sph-1-phosphate, glucosylceramide and lactosylceramide and complex glycosphingolipids, involved in differentiation, apoptosis, and cell cycle arrest [32, 33].

**Fig. 1:**
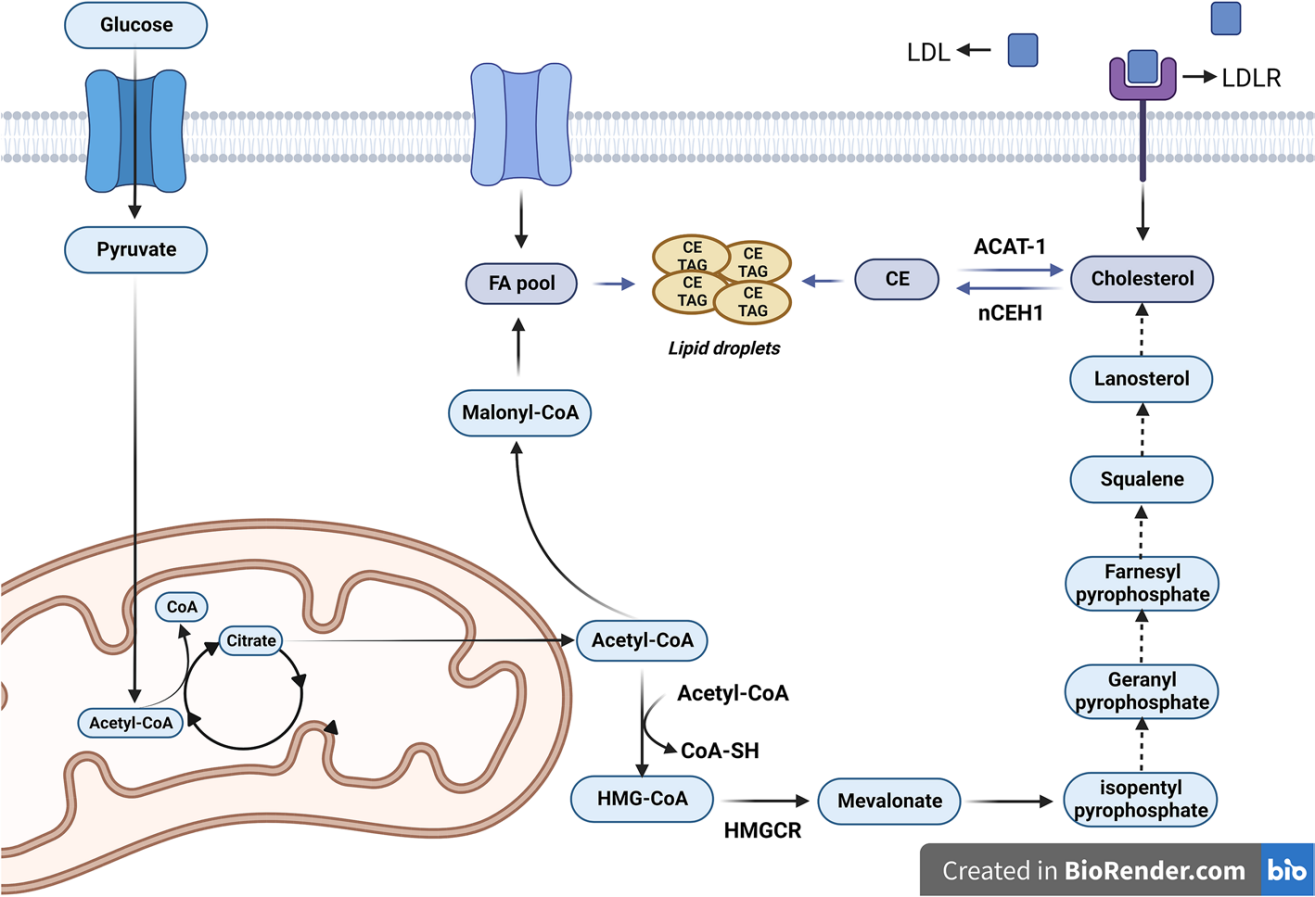
The interplay between glucose and lipid metabolism is evident through the cross-link between these pathways. The glycolytic pathway primarily generates citrate, which serves as a precursor for the production of acetyl-CoA via the tricarboxylic acid (TCA) cycle. Acetyl-CoA is a key molecule involved in the synthesis of fatty acids (FAs) and cholesterol. Excess of free cholesterol, coming from both, exogenous and endogenous source (from the metabolic mevalonate pathway), is esterified by ACAT-1 in the endoplasmic reticulum, and stored as cholesteryl ester (CE) within lipid droplets. Similarly, free FAs can be esterified to form triglycerides (TAG), facilitating their incorporation into lipid droplets. The enzyme neutral cholesteryl ester hydrolase (nCEH) plays a crucial role in the opposite process to ACAT-1. It hydrolyzes CE, breaking it down into free cholesterol and FA. The resulting free cholesterol can then be utilized by the cell for various functions, such as membrane synthesis or signaling pathways. This enzymatic activity helps regulate the balance between stored cholesterol and its free form, as well as the availability of free FAs

Under normal conditions, lipid synthesis occurs in the cytosol through the condensation reaction of two-carbon units, resulting in the formation of acetyl Coenzyme A (acetyl-CoA). Acetyl-CoA is derived from citrate by the enzyme ATP-citrate lyase (ACLY) and is then converted to malonyl-CoA by the enzyme acetyl-CoA carboxylase (ACC) (Fig. 1). FAs are synthesized through a repetitive sequence of reactions catalyzed by the enzyme complex acyl-carrier protein domain of the multifunctional enzyme fatty-acid synthase (FASN). ACC serves as the rate-limiting enzyme in this pathway for FAs synthesis [34]. These reactions generate a fundamental 16-carbon saturated FAs called palmitic acid, which can be further elongated and desaturated to produce a diverse spectrum of saturated and unsaturated FA [35].

The main source of carbon for FAs synthesis is derived from glucose. The initial step takes place in the mitochondrial matrix, where glucose is converted to acetyl-CoA, which is then utilized to synthesize citrate in the mitochondrial tricarboxylic acid (TCA) cycle. Under conditions of high adenosine 5′-triphosphate (ATP)/adenosine 5′-diphosphate (ADP) and nicotinamide adenine dinucleotide (NADH/NAD +), citrate is transported back to the cytosol, where lipids are generated [36].

FAs are incorporated into various types of lipids. They can be converted into diacylglycerides and triacylglycerides via the glycerol phosphate pathway. Moreover, the intermediates of this pathway can be further transformed into different phosphoglycerides, including phosphatidylcholine, phosphatidylethanolamine, phosphatidylglycerol, and phosphatidylserine, which serve as structural components of biological membranes [35]. Additionally, sphingolipids, phosphoinositides, and eicosanoids, which belong to other lipid classes, are also derived from FAs. Eicosanoids, for example, are produced from arachidonic acid through conversion into prostaglandin H2 by cyclooxygenases (COX1 and COX2) or into leukotrienes by leukotriene synthases. Prostaglandin H2 can be further converted into prostaglandin E2 (PGE2), prostacyclin, and thromboxanes [37].

Cholesterol is another significant lipid that plays a vital role in cellular function, as depicted in Fig. 1. It serves as a component of cellular membranes, modulating their fluidity and forming lipid rafts involved in specific signal transduction pathways [38]. Cholesterol is also involved in the synthesis of steroid hormones such as estrogen, progesterone and testosterone. Cells can synthesize cholesterol through the mevalonate pathway or acquire it from extracellular sources via transmembrane receptor proteins, predominantly LDLR. Cholesterol synthesis begins with the condensation of acetyl-CoA and acetoacetyl-CoA to form 3-hydroxy-3-methylglutaryl (HMG)-CoA through the action of HMG-CoA synthase [26]. HMG-CoA is then converted to mevalonate by HMG-CoA reductase (HMGCR), which represents the rate-limiting step in cholesterol synthesis [39]. Subsequently, mevalonate is transformed into isopentyl pyrophosphate, which is further converted to geranyl pyrophosphate. Together with another molecule of isopentyl pyrophosphate, geranyl pyrophosphate is converted to farnesyl pyrophosphate. Two molecules of farnesyl pyrophosphate are then condensed to form squalene through the action of squalene synthase. Squalene undergoes cyclization to produce lanosterol, which is eventually converted into cholesterol through a series of 19 additional reactions [40, 41] (illustrated in Fig. 1). Excessive cholesterol can be detrimental to cells under normal conditions. Hence, it is either converted to cholesterol ester (CE) by acetyl-CoA acetyltransferase (ACAT-1) or to its primary metabolite, 27-hydroxycholesterol (27HC), by CYP27A1. CEs are subsequently stored in lipid droplets, which also sequester excess FAs [42].

It is crucial to highlight that both dietary cholesterol and de novo synthesized cholesterol play a vital role in maintaining homeostasis [43]. It is evident that dysregulated lipid levels in susceptible tissues may be associated with the progression of diseases.

In tumor cells and precancerous tissues, there is an upregulation of endogenous FAs, cholesterol, and cholesterol ester synthesis, which serves to promote cell growth, proliferation, differentiation, and motility of tumor cells [26]. These lipid alterations also interfere with numerous signaling pathways and immunological evasion mechanisms [44]. Therefore, in order to comprehend how lipid metabolism influences the behavior of malignant cells and their ability to evade the immune system, it is imperative to first gain a better understanding of its programming in normal mammalian cells.

### Lipid homeostasis and its role in tumorigenesis

Several studies have shown that the metabolism of proliferating tumor cells is different from normal tissues [45]. In general, two classifications for metabolic changes in cancer are described: metabolic changes at the level of the cancer cell and those secondary to the presence of the tumor (they are manifested in a systemic way). Metabolic changes in the cancer cell were initially observed in the 1920s by Shields Warren who described the hypothesis that cancer lethality is related to tumor cell nutrient depletion. Later, Otto Warburg won the Nobel Prize for demonstrating that cancer cells use glucose more efficiently compared to normal cells, especially simple sugars for energy supply even in the presence of normal levels of oxygen, a phenomenon designated as the Warburg effect. For a long time, researchers emphasize the importance of glucose in the aggressiveness of cancer, however, the change in lipid metabolism is also relevant to promote tumor growth, cell migration, invasion, metastasis, and immune escape. The high rate of cell proliferation requires in addition to an increase in demand for glucose, glutamine, amino acid and, the reprogramming of lipid metabolic [46]. One key aspect of lipid metabolism in tumorigenesis is the upregulation of FAs synthesis. Tumor cells exhibit enhanced de novo FAs synthesis to meet the demands for membrane biogenesis, energy production, and signaling molecule generation. [47]. Its upregulation is driven by the increased activity of key enzymes such as FASN and upregulate monoacylglycerol lipase (MAGL) expression, which controls the intracellular release of FAs [48–50]. Nonetheless, FAs is downregulated in TNBC compared to other BC subtypes, but, interestingly, in TNBC FASN inhibition has anticancer effects both in sensitive cells and in chemoresistant cells, which indicates an indirect role of FASN in TNBC [51–54]. Also, cancer cells can utilize FAs obtained from exogenous sources through the upregulation of adipose triglyceride lipase (ATGL) and hormone-sensitive lipase (HSL) [55, 56]. Together, all these alterations aim the mobilization of FAs and, consequently, tumor development.

Indeed, FA synthesis inhibition in vitro and in vivo xenografts by C75 can reduce cell proliferation and tumor growth [57, 58]. It is known that FASN and ACC have similar expression and localization patterns in normal breast tissue, both restricted to the cell cytoplasm. Intriguingly, the expression of FASN and ACC analyzed by immunohistochemical of in situ and infiltrating carcinoma revealed an increment of their expression in both disease stages [34]. Upregulation of FASN and ACC seems an early event in BC development, and the molecules could be explored as a disease diagnosis markers.

Another study conducted by Magnard et al. [59] have shown an interaction between ACC-alpha (ACCα) and BRCA1 through the BRCA1 C-Terminal (BRCT) domain. BRCA1 mutation may lead to a disruption of the BRCA1-ACCα complex, which, in turn, increases ACCα release and lipogenesis in breast tumor cells, indicating that the ACCα activity could be essential for BC cells survival. Besides, mammary carcinomas present different membrane lipid compositions with higher incorporation of endogenous FAs as palmitate-containing phosphatidylcholine. These alterations are correlated with tumor progression, hormone receptors expression and patient survival [60]. A study of lipidomic conducted in 267 human breast tissues showed that genes related to lipid metabolism were found highly expressed in clinical BC samples [60]. Gene silencing of ACCα, elongation of very long chain fatty acid-like 1 (ELOVL1), FASN, insulin-induced gene 1 (INSIG1), sterol regulatory element-binding protein cleavage-activating protein (SCAP), stearoyl-CoA desaturase (SCD), and thyroid hormone-responsive protein (THRSP) reduced the lipidomic profiles and viability of the BC cells.

MAGL is another key enzyme that also demonstrated involvement in tumor progression through energy supply by FA oxidation and increased malignancy of cancer cells, facilitating proliferation and aggressiveness through the production of signaling lipids including monoacylglycerol, free FAs, and secondary lipid metabolites [50, 61]. Another piece of evidence in support of this was demonstrated by Nomura et al. [50], which have identified increased levels of MAGL levels in aggressive BC cells (231MFP) compared to a nonaggressive model (MCF-7).

In addition, dysregulation in the cholesterol pathway has been associated as a risk factor for several malignancies, including BC. Meta-analysis data indicated that dietary cholesterol was associated with an increased risk to develop BC [62]. BC cells incorporate more LDL-cholesterol, which induces cell lines proliferation, migration, and metastasis [63, 64]. Reduced cell viability and migration in BC cells were observed following membrane cholesterol depletion by cyclodextrin [65]. Membrane fluidity control kept by cholesterol is implicated in drug absorption by cancer cells. Depletion of membrane cholesterol in MCF-7 cells enhanced cellular uptake of doxorubicin [66].

Besides all the links between the lipogenic enzymes and BC development, aggressiveness, immune evasion and progression, we need to consider the heterogeneous nature of the disease, in which the expression and the role of these proteins can vary for each subtype.

### Lipid metabolism and the role in immune escape

The alterations in lipid metabolism in cancer cells also have implications for immune escape mechanisms. Lipids, such as cholesterol and FAs, play a crucial role in modulating immune cell function and inflammatory responses. Dysregulated lipid metabolism in cancer cells can disrupt immune cell activation, infiltration, and effector functions, thereby facilitating immune evasion and impacting multiple aspects of the immune response [44, 67].

In tumors, dysregulated lipid metabolism can lead to an accumulation of free FAs, which can cause oxidative stress by increasing ROS production [68]. This, in turn, can lead to damage to cellular components, including DNA, proteins, and lipids [69]. Furthermore, hypoxia can also promote dysregulated lipid metabolism in cancer cells, which can lead to an accumulation of lipids and cholesterol [70, 71]. This can result in increased oxidative stress, as well as alterations in cell signaling pathways that can promote tumor growth and metastasis [72, 73], and including the secretion of cytokines in the tumor microenvironment [74, 75]. The microenvironment surrounding the tumor is characterized by the presence of different types of cells, including cells of the immune system that assist a pro-inflammatory and pro-tumorigenic environment [76].

An important pathway is activated during scarcity of energy sources, leading to an adenosine monophosphate (AMP) increase in relation to adenosine triphosphate (ATP) levels stimulating the AMP-activated protein kinase (AMPK) [77]. This kinase can suppress the mammalian target signaling pathway of rapamycin (mTOR) which induces cell proliferation and protection against apoptosis [78, 79]. AMPK negatively regulates gluconeogenesis, lipid, and protein synthesis [80]. The performance of AMPK in lipid metabolism is due to its ability to phosphorylate and inhibit Acetyl-CoA carboxylase (ACC), limiting the synthesis of FAs [77, 81].

The mTOR pathway is also regulated through the PI3K/Akt pathway with the inhibition of signaling by phosphatase and the tensin homolog (PTEN) [82]. PTEN regulates PI3K signaling, which controls the lipids’ metabolism stimulating transcription factors of genes involved in FAs biosynthesis and their incorporation into triglycerides and cholesterol [83].

Different alterations in the host immune response have been associated with the PI3K/PTEN/AKT/mTOR pathway [84]. The study by Crane et al. [85] involving breast and prostate cancer cell lines showed that activation of PI3K kinase promotes an immunoresistance increase, especially due to the expression of PD-L1, a negative regulator of T cell function. In another study, the use of PI3K/Akt/mTOR pathway inhibitors in acute myeloid leukemia cells demonstrated the expression regulation of immunological checkpoint ligands and the interference in immune evasion mechanisms of leukemic cells, with a decrease in Programmed cell death protein 1 ligand (PD-L1) expression after treatment with PI3K (idelalisib) and mTOR (everolimus) inhibitors [86].

Programmed cell death protein 1 (PD-1) and its ligand (PD-L1) are important immune regulators, and possible dysfunctions of this axis contribute to tumor metastasis and immune evasion [87, 88]. Lastwika et al. showed that PD-1/PD-L1 expression is induced by the PI3K/Akt/mTOR and AMPK pathway contributing to tumor progression. In this study, the AKT/mTOR pathway stimulated in lung cancer cells caused an increase in PD-L1 expression [89]. Furthermore, in genetically engineered lung cancer mice an mTOR inhibitor combined with a PD-1 antibody decreased tumor growth, increased tumor-infiltrating T cells, and decreased regulatory T cells [89].

Evidence suggests that tumor immune escape may be associated with the AKT-mTOR pathway activation and its role in PD-L1 expression [84]. Detection of PD-L1 and Phosphorylated Akt (p-Akt) in diffuse large B-cell lymphoma (DLBCL) was correlated with clinicopathological features and significantly worse outcomes compared with patients with a single positive expression or both negative expressions [88]. Interestingly, the induction of transcription factors by hypoxia is one of several modulators of PD-1/PD-L1 expression [87], indicating the correlation between the characteristics of malignant tumor cells and mechanisms that induce immune evasion during the metabolism of lipids as a nutrient option.

The immune response against tumors is heavily mediated by key components of the immune system, including T cells, natural killer (NK) cells, dendritic cells (DCs), and macrophages. These immune cells play critical roles in the immune evasion process and contribute to tumor progression and metastasis, although further exploration is required [84].

T lymphocytes, or T cells, can be divided into two major types: cytotoxic T cells (CD8 + T cells) and helper T cells (CD4 + T cells). Both types rely on lipid metabolism and other mechanisms to function properly. Naive T cells have lower energy requirements and primarily rely on mitochondrial function and FAs oxidation. In contrast, effector T cells need to expand their energy sources to meet their increased energy and ATP demands [90].

However, simply having a lipid-enriched TME is not sufficient to fulfill the energy needs of effector T cells. This is because certain T cells, especially CD8 + T cells, lack the ability to synthesize all the necessary enzymes for complete catabolism of different types of FAs. As a result, lipotoxicity increases, leading to exhaustion of CD8 + T cells, impairing their role in immune surveillance [91]. Cholesterol and FA can directly affect the function of immune cells, including T cells, NK cells, and macrophages. Elevated levels of cholesterol and FA in the tumor microenvironment can impair immune cell activation and effector functions. For example, increased cholesterol levels can inhibit T cell receptor signaling and impair T cell proliferation and cytokine production, leading to decreased anti-tumor immune responses. Similarly, FAs can promote the generation of immunosuppressive regulatory T cells (Tregs) and myeloid-derived suppressor cells (MDSCs), which suppress the activity of effector immune cells and dampen immune responses against cancer. Accordingly, it was shown that human BC tissues may release large amounts of free FAs to avoid T cells to eliminate the tumoral ones [92].

Natural killers (NK cells) comprise innate immune cells and induce a strong but non-specific cytolytic response against various physiologically stressed cells, including tumors [93]. FAs and cholesterol are stimuli for T and NK cells and enhance their antitumoral activity. As mentioned by Qin and colleagues, a high-cholesterol-based diet increases the total number of NK cells and positively modulates some of their receptors and effector proteins and molecules, namely granzyme B, perforin [94], interferon-γ (IFN-γ), and tumoral necrosis factor (TNF) [95]. When cholesterol molecules accumulate in NK cells, it is suggested to trigger the antitumoral ability of these cells. However, tumor-infiltrating NK cells usually have their phenotype and function adapted. In many tumors, they are suppressed and no longer act against cancer cells, leading to the promotion of tumoral progression [96]. As confirmed by Jin and collaborators, when NK cells’ cytotoxicity was extinguished by TWS119, an inhibitor of glycogen synthase kinase 3 beta (GSK3β), 4T1 murine BC cells migration was promoted [97].

Both DCs and macrophages are determining cells in the TME, as they present the antigens to other cells and exhibit antitumoral effects as antibody-depending cytotoxicity and release of cytotoxic components [98]. Yet, some phenomena may disestablish the antigen presentation ability of DCs. Dysfunctions in lipid metabolism can lead to lipid accumulation and impairment of MHC expression, thus decreasing antigen presentation to T cells and, as consequence, minimizing its specific response against tumors [99]. Hence, the abrogation of DCs’ antigen presentation may contribute to the ability of cancer cells to evade immune recognition [100]. After the maturation process, macrophages can be characterized by their activation condition. M1 macrophages are activated by the classic pathway, while the M2 ones are alternatively activated [101]. This differentiation occurs because both types play distinct functions. M1 participates in anti-tumor immune response and M2, on the contrary, produces an anti-inflammatory phenotype and secrets many pro-tumor macrophages are influenced to reprogram to the M2 phenotype and mainly rely on FA oxidation as its source of energy [102]. It is important to highlight that TAMs can fulfill their necessity of FA through de novo synthesis from acetyl-CoA [103]. Therefore, Fang and collaborators demonstrated that the growth factor progranulin can promote M2 polarization and PD-L1 expression through the activation of the STAT3 pathway. Their findings suggest that this growth factor may stimulate BC tissues' immune evasion [104]. Consistently, the circular RNA circWWC3 was shown to boost BC progression and tumor immune escape by the promotion of M2 polarization [105].

Exosomes are another example of components that can influence immune evasion and still need to be more investigated. They are lipid vesicles derived from the cell membrane, but their internalization mechanism is not completely elucidated. However, this structure’s involvement in BC carcinogenesis is doubtless [106]. Numerous studies have aimed to demonstrate its key role in immune evasion since this process could explain the ability of BC cells to overcome immune surveillance and become more aggressive and metastatic, worsening the prognosis. As seen by Liu and colleagues, exosomes induced tumor growth in murine models by reducing NK cell activation [107]. Also, Xing and collaborators observed a significant downregulation of X-inactive-specific transcript (XIST) in BC tissues, and this induced exosomal miR-503 release, a microRNA associated with carcinogenesis and angiogenesis [108]. This same study showed that after the exosome release, a greater macrophage polarization from M1 to M2 was noticed because of STAT3 and NFkB signaling pathways modulation, reducing immune response by T cells suppression [108]. Interestingly, exosomal miR-27a-3p induces BC cells' immune evasion through the modulation of macrophage PD-1 expression, by leading MAGI2 to act as an activator of PTEN. Hence, it causes PI3K and, consequently, its signaling pathway’s inactivation. Thus, PTEN-PI3K/AKT signaling pathway influences PD-1 modulation [109]. In addition to these findings, exosomes derived from MDA-MB-231 triple-negative BC and BT-474 luminal B BC cell lines, in a hypoxic environment, perform a potent immunosuppression activity by negatively modulating T cells proliferation through TGF-β and, accordingly, may influence in the immune system evasion process [110].

It is noteworthy that just as different histological subtypes have different metabolic profiles, the TME of each subtype also differs, suggesting a relationship between energy character and immune evasion strategies [111, 112]. In metastatic BC, Boutte' and colleagues reported a significant increase in glycolysis concomitant with the accumulation of MDSC in the TME [113]. However, despite advances, the characterization of the metabolic profile and its impact on the TME is still uncertain and should be investigated in the future.

### Insights on the role of lipid metabolism in specific BC subtypes

As we demonstrated in the overview of the lipid pathways, three important steps are involved in the functionality of the cell: lipid uptake, de novo lipogenesis, and β-oxidation. This way, a cancer cell can develop multiple strategies and changes that run through the uptake, anabolism, and catabolism of lipids to maintain their high energetic need [114]. However, it is worth pointing out that a solid tumor presents itself as a complex system, with the presence of a heterogeneous microenvironment, which contains areas of hypoxia, low pH, necrosis, and nutrient deprivation. In response to these factors, and to meet the demand of different tumor cells, cell metabolism undergoes several changes manifested in a systemic way for each subtype of BC. These peculiarities should be used as a targeted application of metabolic inhibitors that block specific pathways involved in lipid metabolism.

Currently, luminal A, luminal B, HER2-enriched, and basal-like (that also comprises TNBC) are the molecular subtypes frequently recognized at clinical diagnosis. Concerning the luminal group, major differences in metabolites were found for 27-HC [115]. This cholesterol metabolite is part of a group called selective estrogen receptor modulators (SERMs), and acts as an estrogen receptor agonist, enhancing the proliferation of luminal ER + BC cells (MCF-7) in vitro [116] and tumor growth in mice [117]. The 27-HC is metabolized by CYP7B1. Interestingly, ER + breast tumors with elevated expression of CYP7B1 were positively correlated with higher rates of disease-free survival than the ones with low levels of the molecule [115]. It is likely that luminal tumors use 27-HC as fuel for cancer progression.

Apart from classical characteristics already known for TNBC as high expression of proliferation and cell cycle-related genes, they are also distinguished by tumor metabolites. It was shown that lipid metabolite was altered in triple-negative tumors compared to normal adjacent tissue (*p* = 0.001) [118]. On the other hand, this distinct phenotype was not statistically significant for luminal A BC compared to normal tissue [119]. Analysis of 62 breast cancer tissue by Wang et al. [119] revealed that ACLY expression is three times higher in tumor tissue than in normal tissue. Although they did not observe a relation between ACLY expression and ER, PR or HER2 status, a positive correlation was found between p-ACLY expression and ki67 levels (*p* < 0.05), tumor size (*p* < 0.05) and higher histological grade (*p* < 0.05). In contrast with other subtypes, TNBC is often diagnosed with high-grade ductal histology and high Ki-67 [120, 121]. In contrast to this fact, it was revealed that the HER2-enriched subtype expresses more ACLY mRNA than TNBC and luminal tumors [122].

In a recent study by Eiriksson et al. [123], the authors showed the same lipid types synthesized by BC cell lines MCF-7, luminal A subtype, and MDA-MB-231, TNBC subtype as models, mainly the ones called phosphatidylcholines. Compared to the MCF-10A normal tissue cell line, the cancer cells showed higher amounts of this lipid type. It is worth mentioning that a higher amount of phosphatidylcholine was observed in TNBC cells, compared to the luminal A cells [123]. It is hypothesized that the reason for the observation is that phosphatidylcholines participate in the formation processes of structures as HDL and LDL, indispensable for cholesterol metabolism [124]. Therefore, one can speculate that the vesicles responsible for the drugs expelling mechanisms could interfere with the regular transport of the chemotherapy drugs and, thus, could contribute to the development of chemoresistance in BC cells.

Studies have also demonstrated the correlation between cholesterol metabolism and BC subtypes. One of the studies indicated that high levels of the ACAT-1 enzyme, which is responsible for the conversion of cholesterol in CE, are associated with a proliferation mechanism mediated by LDL [125]. Moreover, high levels of oxysterol, a cholesterol metabolite, were found in the plasma of luminal B BC patients [126].

Besides that, a study organized by Catasus and colleagues [127] associated the low-density lipoprotein receptor-related protein 1, also known as apolipoprotein E (ApoE) receptor, with the proliferation and the invasiveness potentials in HER2-enriched and TNBC cell lines.

**Fig. 2:**
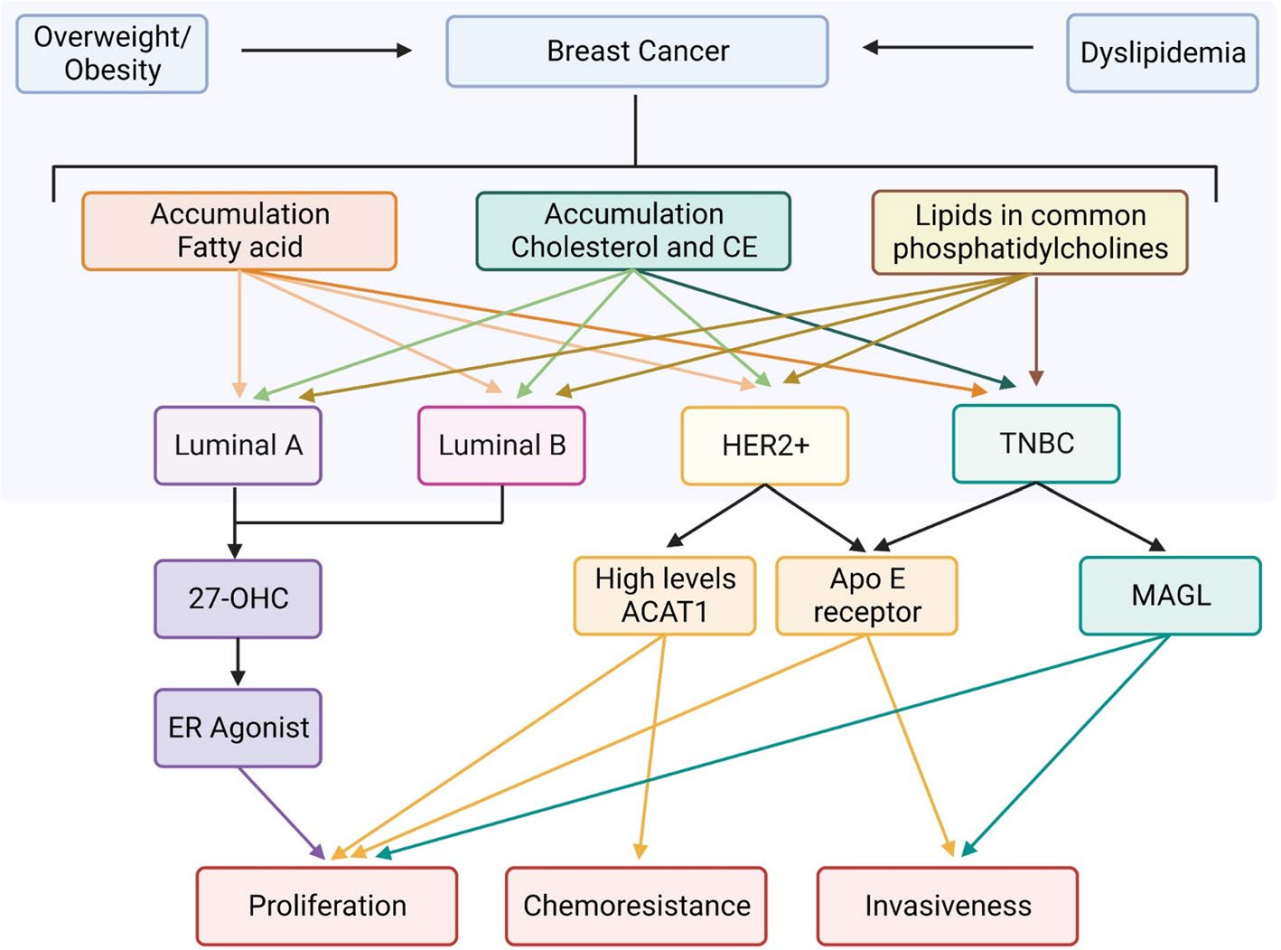
Schematic profiling of integrated lipid metabolism within BC subtypes. Overall studies have suggested that general obesity and dyslipidemia are associated with an increased risk for breast cancer, resulting in accumulation of certain lipids with different magnitude in different types of breast cancer. Fatty acid, cholesterol ester (CE) and phosphatidylcholines have major role in TNBC development. The proliferation of cancer cells in the subtypes are enhanced by different key enzymes. While luminal cells are strongly modulated by the metabolite of cholesterol, 27-OHC (ER agosnist), the TNBC and HER2 + cells share similar profiles being higher in ACAT-1 expression and ApoE content. Being more expressive in TBNC, MAGL can also contribute for an aggressive profile of this cancer

Gonzalo-Calvo and colleagues [128] correlated the accumulation of intratumor CE to the proliferation and aggressive potential of human BC. Therefore, this study demonstrated that HER2-enriched and TNBC patients presented a higher accumulation of intratumor CE compared to the luminal A patients. Each subtype has different metabolic genotypes and phenotypes which influence proliferation, and metastasis capabilities and contribute to chemotherapy resistance (Fig. 2). All these studies and findings suggest that the impact of cholesterol metabolism in BC, especially in the most aggressive subtypes requires more research because it includes a variety of possible therapeutic targets that could improve the treatment of the disease, thus patients’ overall survival and quality of life.

### What does the clinical evidence tell us?

**Fig. 3:**
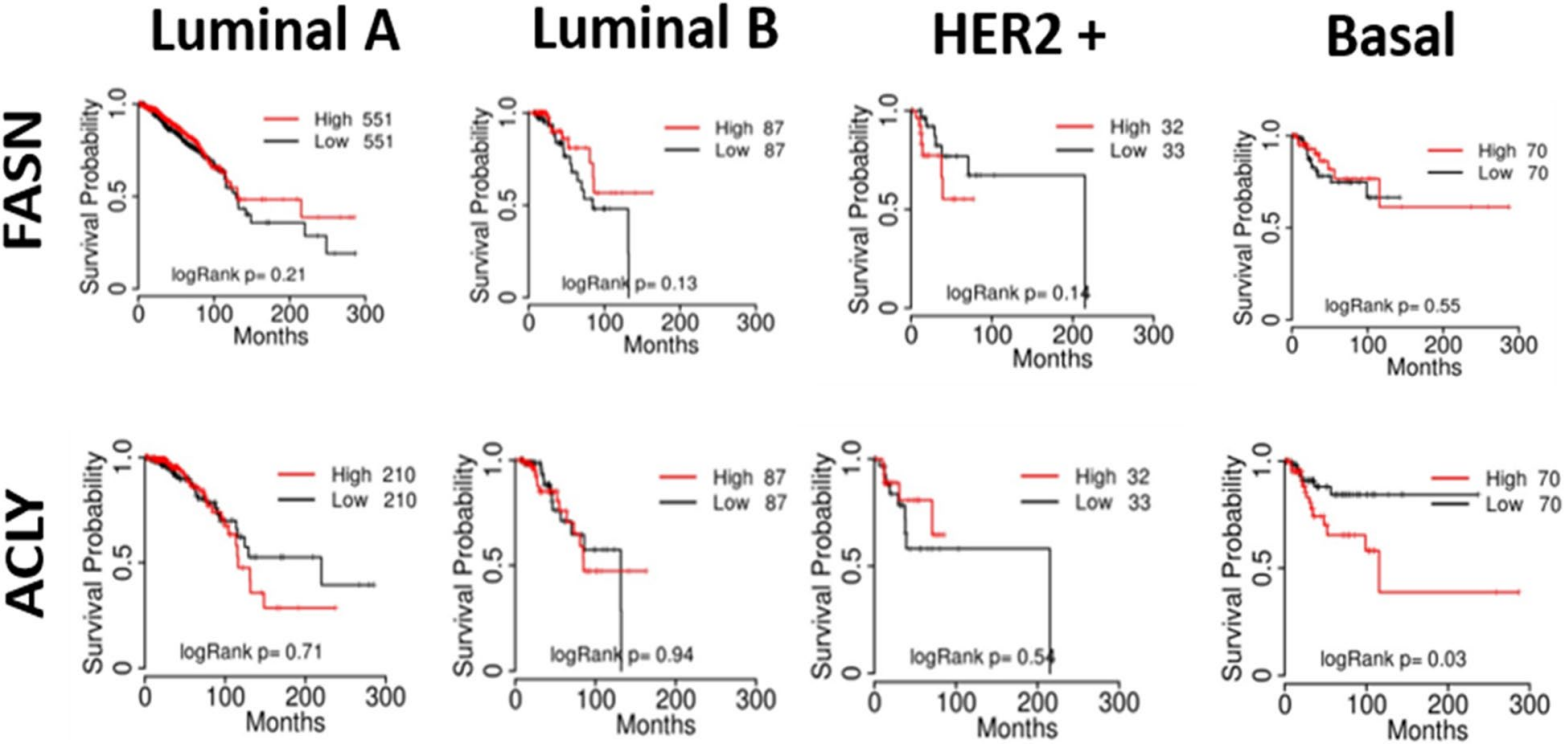
Correlation of key enzymes of FAs metabolism and patient survival in BC subtypes. Kaplan–Meier survival curves were generated using the TCGA website. Patient's survivals probability was compared between two groups divided at median value of protein expression as higher (red) and lower (black) in TCGA data using Kaplan–Meier plotter [129] with best cut-off option

To further explore the linear correlations between the different metabolic profiles and the clinical outcome amongst different subtypes of BC, we collected the data from The Cancer Genome Atlas (TCGA) to compare the survival pattern for BC molecular subtypes with low and high expression of aforementioned lipogenic enzymes. It is worthwhile to inform that basal breast cancer includes the TNBC subtype. FASN expression did not appear to affect patients’ survival probability according to the BC subtype (Fig. 3). Interestingly, despite patients with high and low ACLY expression in the basal group having similar overall survival reaching more than 200 months of follow-up, high ACLY expression reduced 50% of patients’ survival probability (logRank *p* = 0.03), this could suggest that it served as a poor prognostic marker in patients with basal subtype of BC (Fig. 3). However, more specific analyzes must be performed to confirm this phenomenon.

**Fig. 4:**
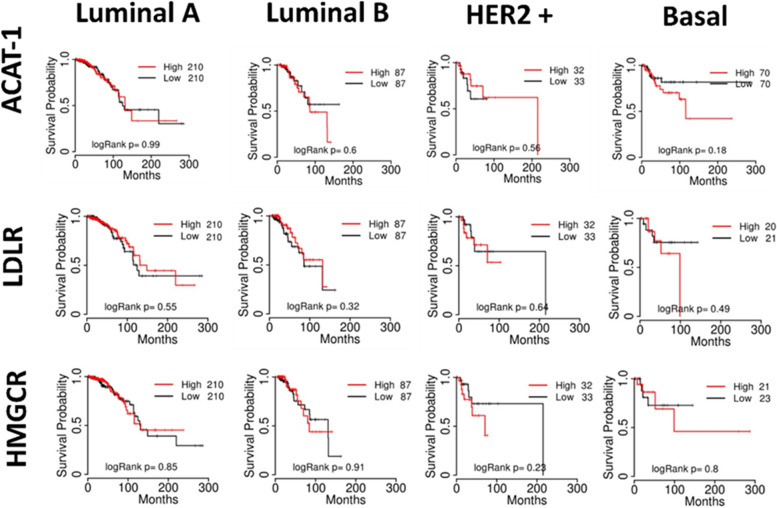
Correlation of key enzymes of cholesterol metabolism and patient survival in BC subtypes. Kaplan–Meier survival curves were generated using the TCGA website. Patient's survivals probability was compared between two groups divided at median value of protein expression as higher (red) and lower (black) in TCGA data using Kaplan–Meier plotter with best cut-off option

Regarding the proteins involved in cholesterol metabolism, our *in-silico* data matches with the studies cited before that demonstrated the upregulation of LDLR and high cholesterol levels in TNBC. Turns out that patients’ survival probability is reduced to 50% after 100 months by ACAT-1 and HMGCR high expression in basal BC but not in other subtypes (Fig. 4). On the other hand, an opposite pattern of patients’ probability survival was seen in relation to LDLR high expression, which dropped dramatically in 100 months (Fig. 4). Taken together, this evidence highlights the complexity behind the expression of the different protein related to lipid metabolism regarding BC subtype.

### Future directions

In the context of highly lethal diseases like TNBC, certain subgroups of patients undergoing new treatments may exhibit improved survival outcomes in the initial years of the disease. Our review demonstrates the involvement of the lipid metabolism pathway in the pathogenesis of BC, particularly highlighting its impact on overall survival probability in different subtypes, with a particular emphasis on the basal group.

However, the perspectives on the metabolic management of BC are still evolving, and there are currently limited studies targeting the specific enzymes involved in BC therapy. One recent completed clinical trial (NCT02595372, concluded in April 2021) explored the use of the proton pump inhibitor omeprazole, known to effectively inhibit FASN, as a potential enhancer of neoadjuvant chemotherapy based on paclitaxel, doxorubicin, and cyclophosphamide.

In addition, an ongoing phase II clinical trial is investigating the use of FASN inhibitors in combination with trastuzumab, paclitaxel, and endocrine therapy for HER2 + BC patients. When considering the role of lipids in BC, cholesterol metabolism appears to play a crucial role in the development of resistance to tamoxifen in luminal A BC [130]. This suggests a plausible hypothesis that cholesterol metabolism may also contribute to the acquisition of chemoresistant phenotypes in other subtypes of BC.

In this regard, HMG-CoA reductase (HMGCR), an enzyme involved in cholesterol synthesis, has been targeted in BC treatment. Numerous ongoing clinical trials (over 40) are evaluating the effects of statins, which inhibit HMGCR, in BC. Among these trials, 14 are specifically assessing the benefits of atorvastatin in combination with conventional chemotherapy, including two phase 2 trials involving TNBC patients (clinicaltrials.gov, NCT03358017; NCT03872388).

While hydroxycitric acid and cucurbitacin B have been tested against ACLY, another enzyme involved in lipid metabolism, these studies were conducted in lung and bladder cancer rather than BC [131]. It has been demonstrated that inhibiting ACLY in combination with conventional chemotherapeutic drugs can enhance treatment efficacy in colorectal cancer [124]. However, there are currently no clinical trials targeting ACLY specifically in BC.

Considering that ACYL, ACAT-1, and LDLR are proteins involved in the activation of FAs and cholesterol from both endogenous and exogenous sources, simultaneous inhibition of these relevant enzymes may lead to a significant anti-tumor response. Therefore, while further functional studies are necessary to better understand the roles of these enzymes and their influence on staging, we suggest giving special attention to tailored therapies for patients with altered expression of ACYL, ACAT-1, and LDLR.

## Conclusion

The evidence compiled in this review underscores the significance of lipid metabolism in normal cellular processes and sheds light on how its dysregulation can contribute to tumorigenesis and immune evasion in BC. These findings serve as a compass for future investigations aimed at unraveling the intricate mechanisms that link disparities in lipid metabolism with variations in prognosis and chemotherapeutic responses in BC. By understanding the nuances of lipid metabolism in BC and its impact on tumor behavior and response to therapies, we can pave the way for developing more effective and tailored treatment strategies for patients.

## Data Availability

The datasets generated and/or analysed during the current study are available in the The Cancer Genome Atlas (TCGA) repository, Breast Cancer (METABRIC, Nature 2012 & Nat Commun 2016) Breast Invasive Carcinoma (TCGA, PanCancer Atlas) available at https://www.cbioportal.org/, and in the ClinicalTrials.gov repository, https://clinicaltrials.gov/ct2/show/NCT02595372, https://clinicaltrials.gov/ct2/show/NCT03358017, https://clinicaltrials.gov/ct2/show/NCT03872388.

## Abbreviations

27HC: 27-Hydroxycholesterol
ACAT: Acyl-CoA cholesterol acyltransferase
ACC: Acetyl-CoA carboxylase
ACLY: ATP-citrate lyase
ADP: Adenosine diphosphate
AKT: Protein kinase B
ApoE: Apolipoprotein E
ATGL: Adipose triglyceride lipase
ATP: Adenosine triphosphate
BC: Breast cancer
BMI: Body mass index
BRCA: Breast cancer gene
BRCT: BRCA1 C-Terminal
CE: Cholesterol ester
CoA: Coenzyme A
COX: Cyclooxygenase
DC: Dendritic cell
ELOVL1: Elongation of very long chain fatty acid-like 1
ER: Estrogen receptor
FAs: Fatty acids
FASN: Fatty-acid synthase
GSK3β: Glycogen synthase kinase 3 beta
HDL: High density lipoprotein
HER2: Human epidermal growth factor receptor 2
HMG: 3-Hydroxy-3-methylglutaryl
HMGCR: 3-Hydroxy-3-methyl-glutaryl-coenzyme A reductase
HSL: Hormone-sensitive lipase
IFN- γ: Interferon-γ
INSIG1: Insulin-induced gene 1
LDL: Low density lipoprotein
LDLR: Low density lipoprotein receptor
MAGI2: Membrane-associated guanylate kinase inverted 2
MAGL: Monoacylglycerol lipase
MHC: Major histocompatibility complex
miRNA: Micro ribonucleic acid
NADH/NAD +: Nicotinamide adenine dinucleotide
NFkB: Nuclear factor kappa-light-chain-enhancer of activated B cells
NK: Natural killer
PD-1: Programmed cell death protein-1
PD-L1: Programmed cell death-ligand 1
PG: Prostaglandin
PI3K: Phosphatidil-inositol-3-kinase
PR: Progesterone receptor
PTEN: Phosphate and tensin homolog
RNA: Ribonucleic acid
SCAP: Sterol regulatory element-binding protein cleavage-activating protein
SCD: Stearoyl-CoA desaturase
SERM: Selective estrogen receptor modulator
STAT3: Signal transducer and activator of transcription 3
TAM: Tumor-associated macrophage
TCA: Tricarboxylic acid
TCGA: The Cancer Genome Atlas
TGF- β: Transforming growth factor beta
THRSP: Thyroid hormone-responsive protein
TIDC: Tumor infiltrated dendritic cell
TME: Tumor microenvironment
TNBC: Triple-negative breast cancer
TNF: Tumoral necrosis factor
XIST: X-inactive-specific transcript

## References

[1] World Health Organization: Breast cancer. 2021. https://www.who.int/news-room/fact-sheets/detail/breast-cancer. Accessed 12 Dec 2022.

[2] Wahba HA, El-Hadaad HA (2015). Current approaches in treatment of triple-negative breast cancer. Cancer Biol Med.

[3] Reddy SM, Barcenas CH, Sinha AK, Hsu L, Moulder SL, Tripathy D, Hortobagyi GN, Valero V (2018). Long-term survival outcomes of triple-receptor negative breast cancer survivors who are disease free at 5 years and relationship with low hormone receptor positivity. Br J Cancer.

[4] Golemis EA, Scheet P, Beck TN (2018). Molecular mechanisms of the preventable causes of cancer in the United States. Genes Dev.

[5] Hahn WC, Bader JS, Braun TP (2021). An expanded universe of cancer targets. Cell.

[6] De Lorenzo A, Gratteri S, Gualtieri P, Cammarano A, Bertucci P, Di Renzo L (2019). Why primary obesity is a disease?. J Transl Med.

[7] Ligibel J (2011). Obesity and breast cancer. Oncology (Williston Park).

[8] Abe R, Kumagai N, Kimura M, Hirosaki A, Nakamura T (1976). Biological characteristics of breast cancer in obesity. Tohoku J Exp Med.

[9] Pajares B, Pollán M, Martín M, Mackey JR, Lluch A, Gavila J, Vogel C, Ruiz-Borrego M, Calvo L, Pienkowski T, Rodríguez-Lescure Á, Seguí MA, Tredan O, Antón A, Ramos M, Cámara Mdel C, Rodríguez-Martín C, Carrasco E, Alba E (2013). Obesity and survival in operable breast cancer patients treated with adjuvant anthracyclines and taxanes according to pathological subtypes: a pooled analysis. Breast Cancer Res.

[10] Dignam JJ, Wieand K, Johnson KA, Fisher B, Xu L, Mamounas EP (2003). Obesity, tamoxifen use, and outcomes in women with estrogen receptor-positive early-stage breast cancer. J Natl Cancer Inst.

[11] Agresti R, Meneghini E, Baili P, Minicozzi P, Turco A, Cavallo I, Funaro F, Amash H, Berrino F, Tagliabue E (2016). Association of adiposity, dysmetabolisms, and inflammation with aggressive breast cancer subtypes: a cross-sectional study. Breast Cancer Res Treat.

[12] Maiti B, Kundranda MN, Spiro TP, Daw HA (2010). The association of metabolic syndrome with triple-negative breast cancer. Breast Cancer Res Treat.

[13] Alves-Bezerra M, Cohen DE (2017). Triglyceride Metabolism in the Liver. Compr Physiol.

[14] Xiang Y, Miao H (2021). Lipid Metabolism in Tumor-Associated Macrophages. Adv Exp Med Biol.

[15] Jiang L, Fang X, Wang H, Li D, Wang X (2018). Ovarian Cancer-Intrinsic Fatty Acid Synthase Prevents Anti-tumor Immunity by Disrupting Tumor-Infiltrating Dendritic Cells. Front Immunol.

[16] Suzuki Y, Tsunoda H, Kimura T, Yamauchi H (2017). BMI change and abdominal circumference are risk factors for breast cancer, even in Asian women. Breast Cancer Res Treat.

[17] Barnard ME, Boeke CE, Tamimi RM (2015). Established breast cancer risk factors and risk of intrinsic tumor subtypes. Biochim Biophys Acta.

[18] Gershuni V, Li YR, Williams AD, So A, Steel L, Carrigan E, Tchou J (2017). Breast cancer subtype distribution is different in normal weight, overweight, and obese women. Breast Cancer Res Treat.

[19] Burkbauer L, Goldbach M, Allison KC, Tchou JC (2020). Obesity and Prognosis in Triple Negative Breast Cancer. J Am Coll Surg.

[20] Davis AA, Kaklamani VG (2012). Metabolic syndrome and triple-negative breast cancer: a new paradigm. Int J Breast Cancer.

[21] Gallagher EJ, Zelenko Z, Neel BA, Antoniou IM, Rajan L, Kase N, LeRoith D (2017). Elevated tumor LDLR expression accelerates LDL cholesterol-mediated breast cancer growth in mouse models of hyperlipidemia. Oncogene.

[22] Kleinfeld AM, Okada C (2005). Free fatty acid release from human breast cancer tissue inhibits cytotoxic T-lymphocyte-mediated killing. J Lipid Res.

[23] Lin R, Zhang H, Yuan Y, He Q, Zhou J, Li S, Sun Y, Li DY, Qiu HB, Wang W, Zhuang Z, Chen B, Huang Y, Liu C, Wang Y, Cai S, Ke Z, He W (2020). Fatty Acid Oxidation Controls CD8+ Tissue-Resident Memory T-cell Survival in Gastric Adenocarcinoma. Cancer Immunol Res.

[24] Fahy E, Subramaniam S, Murphy RC, Nishijima M, Raetz CR, Shimizu T, Spener F, van Meer G, Wakelam MJ, Dennis EA (2009). Update of the LIPID MAPS comprehensive classification system for lipids. J Lipid Res..

[25] Menendez JA, Lupu R (2007). Fatty acid synthase and the lipogenic phenotype in cancer pathogenesis. Nat Rev Cancer.

[26] Song CW, Lee H, Dings RP, Williams B, Powers J, Santos TD, Choi BH, Park HJ (2012). Metformin kills and radiosensitizes cancer cells and preferentially kills cancer stem cells. Sci Rep.

[27] van Meer G, Voelker DR, Feigenson GW (2008). Membrane lipids: where they are and how they behave. Nat Rev Mol Cell Biol.

[28] Ko SH, Jung Y. Energy Metabolism Changes and Dysregulated Lipid Metabolism in Postmenopausal Women. Nutrients. 2021;13(12):4556. Published 2021. 10.3390/nu13124556 10.3390/nu13124556 PMC8704126 34960109

[29] Yang T, Zhao J, Liu F, Li Y (2022). Lipid metabolism and endometrial receptivity. Hum Reprod Update.

[30] Kelly DM, Jones TH. Testosterone: a metabolic hormone in health and disease. J Endocrinol. 2013;217(3):R25-R45. Published 2013:10.1530/JOE-12-0455 10.1530/JOE-12-0455 23378050

[31] Ohanian J, Ohanian V (2001). Lipid second messenger regulation: the role of diacylglycerol kinases and their relevance to hypertension. J Hum Hypertens.

[32] Spaulding SC, Bollag WB. The role of lipid second messengers in aldosterone synthesis and secretion. J Lipid Res. 2022;63(4):100191. 10.1016/j.jlr.2022.100191 10.1016/j.jlr.2022.100191 PMC9020094 35278411

[33] Won JS, Singh AK, Singh I (2007). Lactosylceramide: a lipid second messenger in neuroinflammatory disease. J Neurochem.

[34] Milgraum LZ, Witters LA, Pasternack GR, Kuhajda FP (1997). Enzymes of the fatty acid synthesis pathway are highly expressed in in situ breast carcinoma. Clin Cancer Res.

[35] Baenke F, Peck B, Miess H, Schulze A (2013). Hooked on fat: the role of lipid synthesis in cancer metabolism and tumour development. Dis Model Mech.

[36] Hatzivassiliou G, Zhao F, Bauer DE, Andreadis C, Shaw AN, Dhanak D, Hingorani SR, Tuveson DA, Thompson CB (2005). ATP citrate lyase inhibition can suppress tumor cell growth. Cancer Cell.

[37] Wymann MP, Schneiter R (2008). Lipid signalling in disease. Nat Rev Mol Cell Biol.

[38] Lingwood D, Simons K (2010). Lipid rafts as a membrane-organizing principle. Science.

[39] Chang TY, Chang CC, Ohgami N, Yamauchi Y (2006). Cholesterol sensing, trafficking, and esterification. Annu Rev Cell Dev Biol.

[40] Sharpe LJ, Brown AJ (2013). Controlling cholesterol synthesis beyond 3-hydroxy-3-methylglutaryl-CoA reductase (HMGCR). J Biol Chem.

[41] Billon C, Sitaula S, Burris TP (2016). Inhibition of RORα/γ suppresses atherosclerosis via inhibition of both cholesterol absorption and inflammation. Mol Metab.

[42] Olzmann JA, Carvalho P (2019). Dynamics and functions of lipid droplets. Nat Rev Mol Cell Biol.

[43] Brown MS, Radhakrishnan A, Goldstein JL (2018). Retrospective on Cholesterol Homeostasis: The Central Role of Scap. Annu Rev Biochem.

[44] Yu W, Lei Q, Yang L, et al. Contradictory roles of lipid metabolism in immune response within the tumor microenvironment. J Hematol Oncol. 2021;14(1):187. Published 2021:10.1186/s13045-021-01200-4 10.1186/s13045-021-01200-4 PMC8572421 34742349

[45] Vander Heiden MG, Cantley LC, Thompson CB (2009). Understanding the Warburg effect: the metabolic requirements of cell proliferation. Science.

[46] Fu Y, Zou T, Shen X (2021). Lipid metabolism in cancer progression and therapeutic strategies. MedComm.

[47] Chen WL, Jin X, Wang M, Liu D, Luo Q, Tian H, Cai L, Meng L, Bi R, Wang L, Xie X, Yu G, Li L, Dong C, Cai Q, Jia W, Wei W, Jia L (2020). GLUT5-mediated fructose utilization drives lung cancer growth by stimulating fatty acid synthesis and AMPK/mTORC1 signaling. JCI Insight.

[48] Louie SM, Roberts LS, Nomura DK (2013). Mechanisms linking obesity and cancer. Biochim Biophys Acta.

[49] Zaidi N, Lupien L, Kuemmerle NB, Kinlaw WB, Swinnen JV, Smans K (2013). Lipogenesis and lipolysis: the pathways exploited by the cancer cells to acquire fatty acids. Prog Lipid Res.

[50] Nomura DK, Long JZ, Niessen S, Hoover HS, Ng SW, Cravatt BF (2010). Monoacylglycerol lipase regulates a fatty acid network that promotes cancer pathogenesis. Cell.

[51] Ogrodzinski MP, Bernard JJ, Lunt SY (2017). Deciphering metabolic rewiring in breast cancer subtypes. Transl Res.

[52] Camarda R, Zhou A, Kohnz R (2016). Inhibition of fatty acid oxidation as a therapy for MYC-overexpressing triple-negative breast cancer. Nat Med.

[53] Giró-Perafita A, Palomeras S, Lum DH, Blancafort A, Viñas G, Oliveras G, Pérez-Bueno F, Sarrats A, Welm AL, Puig T (2016). Preclinical Evaluation of Fatty Acid Synthase and EGFR Inhibition in Triple-Negative Breast Cancer. Clin Cancer Res.

[54] Wahdan-Alaswad RS, Cochrane DR, Spoelstra NS, Howe EN, Edgerton SM, Anderson SM, Thor AD, Richer JK. Metformin-induced killing of triple-negative breast cancer cells is mediated by reduction in fatty acid synthase via miRNA-193b. Horm Cancer. 2014 Dec;5(6):374–89. 10.1007/s12672-014-0188-8. Epub 2014 Sep 12. PMID: 25213330; PMCID: PMC4570735. 10.1007/s12672-014-0188-8 PMC4570735 25213330

[55] Balaban S, Shearer RF, Lee LS, van Geldermalsen M, Schreuder M, Shtein HC, Cairns R, Thomas KC, Fazakerley DJ, Grewal T, Holst J, Saunders DN, Hoy AJ (2017). Adipocyte lipolysis links obesity to breast cancer growth: adipocyte-derived fatty acids drive breast cancer cell proliferation and migration. Cancer Metab.

[56] Nieman KM, Kenny HA, Penicka CV, Ladanyi A, Buell-Gutbrod R, Zillhardt MR, Romero IL, Carey MS, Mills GB, Hotamisligil GS, Yamada SD, Peter ME, Gwin K, Lengyel E (2011). Adipocytes promote ovarian cancer metastasis and provide energy for rapid tumor growth. Nat Med.

[57] Pizer ES, Thupari J, Han WF, Pinn ML, Chrest FJ, Frehywot GL, Townsend CA, Kuhajda FP (2000). Malonyl-coenzyme-A is a potential mediator of cytotoxicity induced by fatty-acid synthase inhibition in human breast cancer cells and xenografts. Cancer Res.

[58] Kuhajda FP, Pizer ES, Li JN, Mani NS, Frehywot GL, Townsend CA (2000). Synthesis and antitumor activity of an inhibitor of fatty acid synthase. Proc Natl Acad Sci U S A.

[59] Magnard C, Bachelier R, Vincent A, Jaquinod M, Kieffer S, Lenoir GM, Venezia ND (2002). BRCA1 interacts with acetyl-CoA carboxylase through its tandem of BRCT domains. Oncogene.

[60] Hilvo M, Denkert C, Lehtinen L, Müller B, Brockmöller S, Seppänen-Laakso T, Budczies J, Bucher E, Yetukuri L, Castillo S, Berg E, Nygren H, Sysi-Aho M, Griffin JL, Fiehn O, Loibl S, Richter-Ehrenstein C, Radke C, Hyötyläinen T, Kallioniemi O, Iljin K, Oresic M (2011). Novel theranostic opportunities offered by characterization of altered membrane lipid metabolism in breast cancer progression. Cancer Res.

[61] Ye L, Zhang B, Seviour EG, Tao KX, Liu XH, Ling Y, Chen JY, Wang GB (2011). Monoacylglycerol lipase (MAGL) knockdown inhibits tumor cells growth in colorectal cancer. Cancer Lett.

[62] Li C, Yang L, Zhang D, Jiang W (2016). Systematic review and meta-analysis suggest that dietary cholesterol intake increases risk of breast cancer. Nutr Res.

[63] dos Santos CR, Domingues G, Matias I, Matos J, Fonseca I, de Almeida JM, Dias S (2014). LDL-cholesterol signaling induces breast cancer proliferation and invasion. Lipids Health Dis.

[64] Guan X, Liu Z, Zhao Z, Zhang X, Tao S, Yuan B, Zhang J, Wang D, Liu Q, Ding Y (2019). Emerging roles of low-density lipoprotein in the development and treatment of breast cancer. Lipids Health Dis.

[65] Sharma A, Bandyopadhayaya S, Chowdhury K, Sharma T, Maheshwari R, Das A, Chakrabarti G, Kumar V, Mandal CC. Metformin exhibited anticancer activity by lowering cellular cholesterol content in breast cancer cells. PLoS One. 2019 Jan 9;14(1):e0209435. 10.1371/journal.pone.0209435. 10.1371/journal.pone.0209435 PMC6326520 30625181

[66] Weber P, Wagner M, Schneckenburger H (2013). Cholesterol dependent uptake and interaction of doxorubicin in mcf-7 breast cancer cells. Int J Mol Sci.

[67] Lyssiotis CA, Kimmelman AC (2017). Metabolic Interactions in the Tumor Microenvironment. Trends Cell Biol.

[68] Gao Y, Song Z, Jia L, et al. Self-amplified ROS production from fatty acid oxidation enhanced tumor immunotherapy by atorvastatin/PD-L1 siRNA lipopeptide nanoplexes. Biomaterials. 2022;291:121902. 10.1016/j.biomaterials.2022.121902 10.1016/j.biomaterials.2022.121902 36371945

[69] Nakamura H, Takada K (2021). Reactive oxygen species in cancer: Current findings and future directions. Cancer Sci.

[70] Luo X, Cheng C, Tan Z, et al. Emerging roles of lipid metabolism in cancer metastasis. Mol Cancer. 2017;16(1):76. Published 2017 Apr 11. 10.1186/s12943-017-0646-3 10.1186/s12943-017-0646-3 PMC5387196 28399876

[71] Xia L, Oyang L, Lin J, et al. The cancer metabolic reprogramming and immune response. Mol Cancer. 2021;20(1):28. Published 2021 Feb 5. 10.1186/s12943-021-01316-8. 10.1186/s12943-021-01316-8 PMC7863491 33546704

[72] Roma-Rodrigues C, Mendes R, Baptista PV, Fernandes AR. Targeting Tumor Microenvironment for Cancer Therapy. Int J Mol Sci. 2019;20(4):840. Published 2019 :10.3390/ijms20040840 10.3390/ijms20040840 PMC6413095 30781344

[73] Boutilier AJ, Elsawa SF. Macrophage Polarization States in the Tumor Microenvironment. Int J Mol Sci. 2021;22(13):6995. Published 2021:10.3390/ijms22136995. 10.3390/ijms22136995 PMC8268869 34209703

[74] Engin A (2017). Obesity-associated Breast Cancer: Analysis of risk factors. Adv Exp Med Biol.

[75] Khan S, Shukla S, Sinha S, Meeran SM (2013). Role of adipokines and cytokines in obesity-associated breast cancer: therapeutic targets. Cytokine Growth Factor Rev.

[76] Catalán V, Gómez-Ambrosi J, Rodríguez A, Frühbeck G (2013). Adipose tissue immunity and cancer. Front Physiol.

[77] Wang Q, Liu S, Zhai A, Zhang B, Tian G (2018). AMPK-Mediated Regulation of Lipid Metabolism by Phosphorylation. Biol Pharm Bull.

[78] Herzig S, Shaw RJ (2018). AMPK: guardian of metabolism and mitochondrial homeostasis. Nat Rev Mol Cell Biol.

[79] Inoki K, Kim J, Guan KL (2012). AMPK and mTOR in cellular energy homeostasis and drug targets. Annu Rev Pharmacol Toxicol.

[80] Xiang H, Yang R, Tu J, Xi Y, Yang S, Lv L, Zhai X, Zhu Y, Dong D, Tao X. Metabolic reprogramming of immune cells in pancreatic cancer progression. Biomed Pharmacother. 2023 Jan;157:113992. 10.1016/j.biopha.2022.113992. 10.1016/j.biopha.2022.113992 36395610

[81] Lepropre S, Kautbally S, Octave M, Ginion A, Onselaer MB, Steinberg GR, Kemp BE, Hego A, Wéra O, Brouns S, Swieringa F, Giera M, Darley-Usmar VM, Ambroise J, Guigas B, Heemskerk J, Bertrand L, Oury C, Beauloye C, Horman S (2018). AMPK-ACC signaling modulates platelet phospholipids and potentiates thrombus formation. Blood.

[82] Porta C, Paglino C, Mosca A (2014). Targeting PI3K/Akt/mTOR Signaling in Cancer. Front Oncol.

[83] Chen CY, Chen J, He L, Stiles BL (2018). PTEN: Tumor Suppressor and Metabolic Regulator. Front Endocrinol (Lausanne).

[84] Yang L, Li A, Lei Q, Zhang Y (2019). Tumor-intrinsic signaling pathways: key roles in the regulation of the immunosuppressive tumor microenvironment. J Hematol Oncol.

[85] Crane CA, Panner A, Murray JC, Wilson SP, Xu H, Chen L, Simko JP, Waldman FM, Pieper RO, Parsa AT (2009). PI(3) kinase is associated with a mechanism of immunoresistance in breast and prostate cancer. Oncogene.

[86] Taghiloo S, Norozi S, Asgarian-Omran H (2022). The Effects of PI3K/Akt/mTOR Signaling Pathway Inhibitors on the Expression of Immune Checkpoint Ligands in Acute Myeloid Leukemia Cell Line. Iran J Allergy Asthma Immunol.

[87] Ni JM, Ni AP (2018). Landscape of PD-1/PD-L1 Regulation and Targeted Immunotherapy. Chin Med Sci J.

[88] Dong L, Lv H, Li W, Song Z, Li L, Zhou S, Qiu L, Qian Z, Liu X, Feng L, Meng B, Fu K, Wang X, Pan-Hammarström Q, Wang P, Wang X, Zhang H (2016). Co-expression of PD-L1 and p-AKT is associated with poor prognosis in diffuse large B-cell lymphoma via PD-1/PD-L1 axis activating intracellular AKT/mTOR pathway in tumor cells. Oncotarget.

[89] Lastwika KJ, Wilson W, Li QK, Norris J, Xu H, Ghazarian SR, Kitagawa H, Kawabata S, Taube JM, Yao S, Liu LN, Gills JJ, Dennis PA (2016). Control of PD-L1 Expression by Oncogenic Activation of the AKT-mTOR Pathway in Non-Small Cell Lung Cancer. Cancer Res.

[90] Gubser PM, Bantug GR, Razik L, Fischer M, Dimeloe S, Hoenger G, Durovic B, Jauch A, Hess C (2013). Rapid effector function of memory CD8+ T cells requires an immediate-early glycolytic switch. Nat Immunol.

[91] Manzo T, Prentice BM, Anderson KG, Raman A, Schalck A, Codreanu GS, Nava Lauson CB, Tiberti S, Raimondi A, Jones MA (2020). Accumulation of long-chain fatty acids in the tumor microenvironment drives dysfunction in intrapancreatic CD8+ T cells. J Exp Med.

[92] Kleinfeld AM, Okada C (2005). Free fatty acid release from human breast cancer tissue inhibits cytotoxic T-lymphocyte-mediated killing. J Lipid Res.

[93] Paul S, Lal G (2017). The Molecular Mechanism of Natural Killer Cells Function and Its Importance in Cancer Immunotherapy. Front Immunol.

[94] Qin WH, Yang ZS, Li M, Chen Y, Zhao XF, Qin YY, Song JQ, Wang BB, Yuan B, Cui XL (2020). High serum levels of cholesterol increase antitumor functions of nature killer cells and reduce growth of liver tumors in mice. Gastroenterology.

[95] Xu J, Niu T (2020). Natural killer cell-based immunotherapy for acute myeloid leukemia. J Hematol Oncol.

[96] Arianfar E, Shahgordi S, Memarian A (2021). Natural Killer Cell Defects in Breast Cancer: A Key Pathway for Tumor Evasion. Int Rev Immunol.

[97] Jin F, Wu Z, Hu X (2019). The PI3K/Akt/GSK-3β/ROS/eIF2B pathway promotes breast cancer growth and metastasis via suppression of NK cell cytotoxicity and tumor cell susceptibility. Cancer Biol Med.

[98] Yu Y, Gao L, Wang Y, et al. A Forgotten Corner in Cancer Immunotherapy: The Role of Lipids. *Front Oncol*. 2021;11:751086. Published 2021:10.3389/fonc.2021.751086 10.3389/fonc.2021.751086 PMC8551635 34722305

[99] Herber DL, Cao W, Nefedova Y, Novitskiy SV, Nagaraj S, Tyurin VA, Corzo A, Cho HI, Celis E, Lennox B (2010). Lipid accumulation and dendritic cell dysfunction in cancer. Nat Med.

[100] Jhunjhunwala S, Hammer C, Delamarre L (2021). Antigen presentation in cancer: insights into tumour immunogenicity and immune evasion. Nat Rev Cancer.

[101] Martinez FO, Gordon S. The M1 and M2 paradigm of macrophage activation: time for reassessment. *F1000Prime Rep*. 2014;6:13. Published 2014:10.12703/P6-13 10.12703/P6-13 PMC3944738 24669294

[102] Su P, Wang Q, Bi E, Ma X, Liu L, Yang M, Qian J, Yi Q (2020). Enhanced lipid accumulation and metabolism are required for the differentiation and activation of tumor-associated macrophages. Cancer Res.

[103] Arts RJ, Plantinga TS, Tuit S, Ulas T, Heinhuis B, Tesselaar M, Sloot Y, Adema GJ, Joosten LA, Smit JW (2016). Transcriptional and metabolic reprogramming induce an inflammatory phenotype in non-medullary thyroid carcinoma-induced macrophages. Oncoimmunology.

[104] Fang W, Zhou T, Shi H, et al. Progranulin induces immune escape in breast cancer via up-regulating PD-L1 expression on tumor-associated macrophages (TAMs) and promoting CD8+ T cell exclusion [published correction appears in J Exp Clin Cancer Res. 2022 Mar 12;41(1):93]. J Exp Clin Cancer Res. 2021;40(1):4. Published 2021:10.1186/s13046-020-01786-6 10.1186/s13046-020-01786-6 PMC7780622 33390170

[105] Zheng Y, Ren S, Zhang Y, et al. Circular RNA circWWC3 augments breast cancer progression through promoting M2 macrophage polarization and tumor immune escape via regulating the expression and secretion of IL-4. Cancer Cell Int. 2022;22(1):264. Published 2022:10.1186/s12935-022-02686-9 10.1186/s12935-022-02686-9 PMC9396792 35996149

[106] Giordano C, La Camera G, Gelsomino L, et al. The Biology of Exosomes in Breast Cancer Progression: Dissemination, Immune Evasion and Metastatic Colonization. Cancers (Basel). 2020;12(8):2179. Published 2020:10.3390/cancers12082179 10.3390/cancers12082179 PMC7465598 32764376

[107] Liu C, Yu S, Zinn KR, Wang J, Zhang L, Jia Y, Kappes JC, Barnes S, Kimberly RP, Grizzle WE (2006). os exossomos de carcinoma mamário murino promovem o crescimento tumoral pela supressão da função das células NK. J Immunol.

[108] Xing F, Liu Y, Wu SY, Wu K, Sharma S, Mo YY, Feng J, Sanders S, Jin G, Singh R, Vidi PA, Tyagi A, Chan MD, Ruiz J, Debinski W, Pasche BC, Lo HW, Metheny-Barlow LJ, D'Agostino RB Jr, Watabe K. Loss of XIST in Breast Cancer Activates MSN-c-Met and Reprograms Microglia via Exosomal miRNA to Promote Brain Metastasis. Cancer Res. 2018 Aug 1;78(15):4316–4330.10.1158/0008-5472.CAN-18-1102. Epub 2018 Jul 19. Erratum in: Cancer Res. 2021;81(21):5582.

[109] Yao P, Ni Y, Liu C (2020). Long Non-Coding RNA 691 Regulated PTEN/PI3K/AKT Signaling Pathway in Osteosarcoma Through miRNA-9-5p. Onco Targets Ther.

[110] Rong L, Li R, Li S, Luo R (2015). Imunossupressão de células de câncer de mama mediada pela transformação do fator de crescimento β em exossomos de células cancerígenas. Oncol Deixe.

[111] Onkar SS, Carleton NM, Lucas PC, Bruno TC, Lee AV, Vignali DAA, Oesterreich S. The Great Immune Escape: Understanding the Divergent Immune Response in Breast Cancer Subtypes. Cancer Discov. 2022:OF1-OF18.: 10.1158/2159-8290.CD-22-0475. 10.1158/2159-8290.CD-22-0475 PMC9833841 36620880

[112] Dias AS, Almeida CR, Helguero LA, Duarte IF (2019). Metabolic crosstalk in the breast cancer microenvironment. Eur J Cancer.

[113] Boutte´ AM, McDonald WH, Shyr Y, Yang L, Lin PC. Characterization of the MDSC proteome associated with metastatic murine mammary tumors using label-free mass spectrometry and shotgun proteomics. PLoS One 2011;6:e22446. 10.1371/journal.pone.0022446. 10.1371/journal.pone.0022446 PMC3154190 21853032

[114] Yoon S, Lee MY, Park SW, Moon JS, Koh YK, Ahn YH, Park BW, Kim KS (2007). Up-regulation of acetyl-CoA carboxylase alpha and fatty acid synthase by human epidermal growth factor receptor 2 at the translational level in breast cancer cells. J Biol Chem.

[115] DuSell CD, Umetani M, Shaul PW, Mangelsdorf DJ, McDonnell DP (2008). 27-hydroxycholesterol is an endogenous selective estrogen receptor modulator. Mol Endocrinol.

[116] Cruz P, Torres C, Ramírez ME, Epuñán MJ, Valladares LE, Sierralta WD (2010). Proliferation of human mammary cancer cells exposed to 27-hydroxycholesterol. Exp Ther Med.

[117] Wu Q, Ishikawa T, Sirianni R, Tang H, McDonald JG, Yuhanna IS, Thompson B, Girard L, Mineo C, Brekken RA, Umetani M, Euhus DM, Xie Y, Shaul PW (2013). 27-Hydroxycholesterol promotes cell-autonomous. ER-positive breast cancer growth Cell Rep.

[118] Tayyari F, Gowda GAN, Olopade OF, Berg R, Yang HH, Lee MP, Ngwa WF, Mittal SK, Raftery D, Mohammed SI (2018). Metabolic profiles of triple-negative and luminal A breast cancer subtypes in African-American identify key metabolic differences. Oncotarget.

[119] Wang J, Li M, Chen D, Nie J, Xi Y, Yang X, Chen Y, Yang Z (2017). Expression of C-myc and β-catenin and their correlation in triple negative breast cancer. Minerva Med.

[120] Soliman, N. A., & Yussif, S. M. (2016). Ki-67 as a prognostic marker according to breast cancer molecular subtype. Cancer Biology and Medicine, 13(4), 496–504. 10.20892/j.issn.2095-3941.2016.0066 10.20892/j.issn.2095-3941.2016.0066 PMC5250608 28154782

[121] Pan Y, Yuan Y, Liu G, Wei Y. P53 and Ki-67 as prognostic markers in triple-negative breast cancer patients. PLoS One. 2017 Feb 24;12(2):e0172324. doi: 10.1371/journal.pone.0172324. 10.1371/journal.pone.0172324 PMC5325264 28235003

[122] Giró-Perafita A, Sarrats A, Pérez-Bueno F, Oliveras G, Buxó M, Brunet J, Viñas G, Miquel TP (2017). Fatty acid synthase expression and its association with clinico-histopathological features in triple-negative breast cancer. Oncotarget.

[123] Eiriksson FF, Nøhr MK, Costa M, Bödvarsdottir SK, Ögmundsdottir HM, Thorsteinsdottir M. Lipidomic study of cell lines reveals differences between breast cancer subtypes. PLoS One. 2020 Apr 14;15(4):e0231289. 10.1371/journal.pone.0231289. 10.1371/journal.pone.0231289 PMC7156077 32287294

[124] Cotte AK, Aires V, Fredon M, Limagne E, Derangère V, Thibaudin M, Humblin E, Scagliarini A, de Barros JP, Hillon P, Ghiringhelli F, Delmas D (2018). Lysophosphatidylcholine acyltransferase 2-mediated lipid droplet production supports colorectal cancer chemoresistance. Nat Commun.

[125] Antalis CJ, Arnold T, Rasool T, Lee B, Buhman KK, Siddiqui RA (2010). High ACAT1 expression in estrogen receptor negative basal-like breast cancer cells is associated with LDL-induced proliferation. Breast Cancer Res Treat.

[126] Kloudova-Spalenkova A, Ueng YF, Wei S, Kopeckova K, Peter Guengerich F, Soucek P. Plasma oxysterol levels in luminal subtype breast cancer patients are associated with clinical data. J Steroid Biochem Mol Biol. 2020 Mar;197:105566. 10.1016/j.jsbmb.2019.105566. 10.1016/j.jsbmb.2019.105566 PMC7015808 31874216

[127] Catasus L, Gallardo A, Llorente-Cortes V, Escuin D, Muñoz J, Tibau A, Peiro G, Barnadas A, Lerma E (2011). Low-density lipoprotein receptor-related protein 1 is associated with proliferation and invasiveness in Her-2/neu and triple-negative breast carcinomas. Hum Pathol.

[128] de Gonzalo-Calvo D, López-Vilaró L, Nasarre L, Perez-Olabarria M, Vázquez T, Escuin D, Badimon L, Barnadas A, Lerma E, Llorente-Cortés V (2015). Intratumor cholesteryl ester accumulation is associated with human breast cancer proliferation and aggressive potential: a molecular and clinicopathological study. BMC Cancer.

[129] Győrffy B. Survival analysis across the entire transcriptome identifies biomarkers with the highest prognostic power in breast cancer. Comput Struct Biotechnol J. 2021;19:4101–4109. Published 2021:10.1016/j.csbj.2021.07.014 10.1016/j.csbj.2021.07.014 PMC8339292 34527184

[130] Hultsch S, Kankainen M, Paavolainen L, Kovanen RM, Ikonen E, Kangaspeska S, Pietiäinen V, Kallioniemi O (2018). Association of tamoxifen resistance and lipid reprogramming in breast cancer. BMC Cancer.

[131] Guais A, Baronzio G, Sanders E, Campion F, Mainini C, Fiorentini G, Montagnani F, Behzadi M, Schwartz L, Abolhassani M (2012). Adding a combination of hydroxycitrate and lipoic acid (METABLOC™) to chemotherapy improves effectiveness against tumor development: experimental results and case report. Invest New Drugs.

